# Benchmarking the Effectiveness and Accuracy of Multiple Mitochondrial DNA Variant Callers: Practical Implications for Clinical Application

**DOI:** 10.3389/fgene.2022.692257

**Published:** 2022-03-08

**Authors:** Eddie K. K. Ip, Michael Troup, Colin Xu, David S. Winlaw, Sally L. Dunwoodie, Eleni Giannoulatou

**Affiliations:** ^1^ Victor Chang Cardiac Research Institute, Sydney, NSW, Australia; ^2^ St. Vincent’s Clinical School, Sydney, NSW, Australia; ^3^ School of Computer Science and Engineering, Sydney, NSW, Australia; ^4^ Cardiothoracic Surgery, Cincinnati Children’s Hospital Medical Centre, Heart Institute, Cincinnati, OH, United States

**Keywords:** whole-genome sequencing, mitochondrial DNA, variant‐caller, homoplasmic, heteroplasmic, benchmarking

## Abstract

Mitochondrial DNA (mtDNA) mutations contribute to human disease across a range of severity, from rare, highly penetrant mutations causal for monogenic disorders to mutations with milder contributions to phenotypes. mtDNA variation can exist in all copies of mtDNA or in a percentage of mtDNA copies and can be detected with levels as low as 1%. The large number of copies of mtDNA and the possibility of multiple alternative alleles at the same DNA nucleotide position make the task of identifying allelic variation in mtDNA very challenging. In recent years, specialized variant calling algorithms have been developed that are tailored to identify mtDNA variation from whole-genome sequencing (WGS) data. However, very few studies have systematically evaluated and compared these methods for the detection of both homoplasmy and heteroplasmy. A publicly available synthetic gold standard dataset was used to assess four mtDNA variant callers (Mutserve, mitoCaller, MitoSeek, and MToolBox), and the commonly used Genome Analysis Toolkit “best practices” pipeline, which is included in most current WGS pipelines. We also used WGS data from 126 trios and calculated the percentage of maternally inherited variants as a metric of calling accuracy, especially for homoplasmic variants. We additionally compared multiple pathogenicity prediction resources for mtDNA variants. Although the accuracy of homoplasmic variant detection was high for the majority of the callers with high concordance across callers, we found a very low concordance rate between mtDNA variant callers for heteroplasmic variants ranging from 2.8% to 3.6%, for heteroplasmy thresholds of 5% and 1%. Overall, Mutserve showed the best performance using the synthetic benchmark dataset. The analysis of mtDNA pathogenicity resources also showed low concordance in prediction results. We have shown that while homoplasmic variant calling is consistent between callers, there remains a significant discrepancy in heteroplasmic variant calling. We found that resources like population frequency databases and pathogenicity predictors are now available for variant annotation but still need refinement and improvement. With its peculiarities, the mitochondria require special considerations, and we advocate that caution needs to be taken when analyzing mtDNA data from WGS data.

## 1 Introduction

The mitochondrion is an organelle in eukaryotic cells responsible for manufacturing most of the cell’s energy. It possesses its own double-stranded circular genome of 16,569 nucleotides, which encodes for the 12S and 16S rRNAs, 22 tRNAs, and 13 polypeptides (Anderson et al., 1981; Taanman 1999). Typically, mitochondrial DNA (mtDNA) is only inherited from the mother because the mitochondria from the sperm cell are usually destroyed by the egg shortly after fertilization; a phenomenon known as a matrilineal inheritance (Sutovsky et al., 1999). There are multiple copies (∼100–10,000 copies) of mtDNA in a mitochondrion and many mitochondria per somatic cell. With a mutation rate of more than a hundred folds higher than the nuclear genome, the DNA sequence at any base of the mtDNA genome may differ between mtDNA copies (Kogelnik et al., 1998; Marcelino and Thilly 1999; Just et al., 2015; Stewart and Chinnery 2015).

The genetic variation in mtDNA is classified into two categories: 1) homoplasmic variants, which occur when an alternative allele appears in all copies of the mtDNA genome, and are expected to be inherited from the mother, and 2) heteroplasmic variants, which occur when an alternative allele is only present in some copies of the mtDNA genome (Taylor and Turnbull 2005; Stewart and Chinnery 2015). Heteroplasmic variants are often sporadic, appearing throughout an individual’s lifetime, but can also be inherited from the mother and can have an allele frequency as low as 1% (Guo et al., 2013a; Rebolledo-Jaramillo et al., 2014). Mitochondrial DNA variants are known to contribute to human disease with varying severity, from rare, highly penetrant mutations causing monogenic disorders that often affect the nervous system, muscles, heart, and endocrine organs, to mutations with milder contributions to phenotypes (Taylor and Turnbull 2005). The small size of the mitochondrial genome compared to the full human genome might imply that mtDNA variant calling is a straightforward task. However, the large number of copies of mtDNA and the possibility of multiple alternative alleles at the same DNA nucleotide position make the task of identifying allelic variation in mtDNA much more challenging. In addition, the human mitochondrial genome contains a total of 31 repeats that are more than 12 bp in length (Phillips et al., 2017). The presence of repeats when short read sequencing data are used adds an additional challenge to this task.

Whole-genome sequencing (WGS) is becoming the default sequencing option for most research studies, fueled by the drop in cost to under $USD1000 for human DNA (Wetterstrand 2019). A surprising benefit of WGS for a human sample is that because of the high number of mitochondria that exists in human cells, the smaller mitochondrial genome is also captured and sequenced, with coverage of >1,000 reads typically achieved. As a result, both homoplasmic and heteroplasmic variants can be detected using WGS. Homoplasmic variants are expected to be found across all sequencing reads at a specific position, while heteroplasmic variants would occur in a percentage of the sequencing reads. This percentage of mtDNA variant allele frequency (VAF) can be as low as 1% and can be often hard to distinguish from technical errors (Rebolledo-Jaramillo et al., 2014; Guo et al., 2013b). Given the large number of copies of mtDNA, a variant with 1% frequency can still be present in thousands of mtDNA copies, especially in specific tissues such as in oocytes.

In recent years, specialized variant calling algorithms have been developed that are tailored to identify mtDNA variation from WGS data (Weissensteiner et al., 2016; Ding et al., 2015; Guo et al., 2013a; Calabrese et al., 2014). These methods utilize the high-depth of coverage of the mitochondrial genome and go beyond the three discrete genotype categories of the nuclear DNA to identify both homoplasmic and heteroplasmic variants that might only have occurred in a fraction of all the mtDNA copies (Just et al., 2015). However, as it has been shown for other algorithms tailored for the nuclear DNA or for somatic variant identification (Hwang et al., 2015; Chen et al., 2019; Wang et al., 2019; Chen et al., 2020), variant calling algorithms can often exhibit low concordance and can suffer from many false positives. Accurate identification of mtDNA variation will facilitate disease-sequencing studies that currently often ignore the mitochondrial genome.

In this study, we provide a systematic evaluation and benchmarking of multiple mtDNA variant calling algorithms. We evaluate five variant callers, Mutserve (Weissensteiner et al., 2016), mitoCaller (Ding et al., 2015), MitoSeek (Guo et al., 2013b), MToolBox (Calabrese et al., 2014), and a mtDNA-tailored modification of GATK HaplotypeCaller (DePristo et al., 2011) using a synthetic gold-standard two-person mtDNA mixture sequenced using Illumina MiSeq, and 300 mtDNA samples forming 126 trios, which were sequenced using Illumina HiSeq X Ten. In addition, all resulting variants from the trio analysis were annotated using control population frequencies and pathogenicity prediction databases. Our results provide useful insights for researchers and clinicians analyzing mitochondrial genomes obtained from WGS experiments for disease studies.

## 2 Materials and Methods

### 2.1 Synthetic Gold-Standard Dataset

We utilized a recently constructed heteroplasmy benchmark dataset developed by Fazzini et al. (2021). This benchmark dataset contains 27 expected artificial heteroplasmic sites (which is the result of the mixture of the haplotypes H1c6 and U5a2e in reference to the mtDNA reference genome) and 6 homoplasmic variants and 3 private mutations. The M4 mixture of 1:100 corresponding to heteroplasmy levels of 1% was used for the algorithms’ evaluation.

#### 2.1.1 Sample Collection and Sequencing

As described by Fazzini et al. (2021), the samples were sequenced on Illumina MiSeq with three different Taq polymerases [Clontech LA Advantage (Clontech/CLAA), LongAmp Taq Polymerase (NEB), and Herculase Fusion (HERK)] and different DNA extraction protocols (using PCR products and using total DNA, i.e., PCR free). One sample per library preparation method and DNA extraction method was selected from Fazzini et al. (2021) by selecting the samples with the highest coverage. The following samples were used: M4-Clontech_S211, M4-Herk_S151, M4-NEB_S241, M4-PCR-Clontech_S191, M4-PCR-Herk_S141, and M4-PCR-NEB_S291.

#### 2.1.2 Alignment to a Reference Genome

The genomic data were obtained in the form of FASTQ files from the provided link: https://zenodo.org/record/3991749#.YNKDNZMzaWg. The data were aligned to the revised Cambridge Reference Sequence (rCRS) mitochondrial sequence NC_012920.1 using the Burrows–Wheeler Aligner (BWA) 0.7.17 (Li and Durbin 2009) to create mtDNA binary alignment map (BAM) files. Duplicates were marked using The Genome Analysis Toolkit (GATK) v4.2.0.0 (DePristo et al., 2011).

#### 2.1.3 Mitochondrial Variant Calling

Four mtDNA-specific variant callers (Mutserve v1.1.17 (Weissensteiner et al., 2016), mitoCaller v1.0 (Ding et al., 2015), MitoSeek v.1.3 (Guo et al., 2013a), and MToolBox v1.2 (Calabrese et al., 2014)) were used for our evaluation, as well as the GATK Haplotypecaller v4.2.0.0 pipeline (DePristo et al., 2011) (Table 1). Variant calling for the synthetic dataset was performed at standardized heteroplasmy detection evaluation at 1% threshold. For mitoCaller and GATK Haplotypecaller, which did not have a user parameter to define a heteroplasmy variant detection threshold, custom scripts were used to filter returned heteroplasmic variants that met the experimental threshold of 1%.

**TABLE 1:** A descriptive summary of mtDNA variant callers selected for comparative analyses.

Features	Mutserve v1.1.17	MitoSeek v1.3	mitoCaller v1.0	MToolBox v1.2	GATK v3.7 and v4.2- HaplotypeCaller
	Version from 19/02/19	Version from 13/05/2019	Version from 16/01/2018	Version from 15/11/2021	Versions from 12/12/2016 and 18/0/2021
Process Location	Local, command line	Local, command line	Local, command line	Local, command line	Local, command line
Input File format	BAM	BAM	BAM	BAM	BAM
Mitochondrial alignment	rCRS mitochondrial reference	rCRS mitochondrial reference	“double alignment” strategy, with rCRS mitochondrial reference and a shifted rCRS reference	RSRS and rCRS mitochondrial reference	rCRS mitochondrial reference
Heteroplasmic variant detection	Yes	Yes	Yes	Yes	Yes
Homoplasmic variant detection	Yes	No	Yes	Yes	Yes
Default heteroplasmic threshold	1%	5%	Based on likelihood model	20%	Based on likelihood model
Altering heteroplasmic threshold	--level parameter	-hp parameter	a custom script for threshold detection was used in the study	hf_min parameter in config file	a custom script for threshold detection was used in the study
Description of the variant-calling algorithm	Maximum likelihood model for detecting heteroplasmic variants	One-tail Fisher’s exact test for detecting heteroplasmic variants	Maximum likelihood-based model for both heteroplasmic and homoplasmic variants detection	Identification of mismatches in the newly assembled mitochondrial genome (or differences in the SAM CIGAR string for INDELs)	Bayesian model for both heteroplasmic and homoplasmic variants detection

##### 2.1.3.1 Mutserve

Mutserve is a local version of the scalable web server “mtDNA-server,” which was released in 2016 (Weissensteiner et al., 2016). It has been used in variant identification studies for diseases like congenital lactic acidosis and gastric cancer (Bravo-Alonso et al., 2019; Cavalcante et al., 2019). Mutserve performs internal quality control by excluding mitochondrial hotspots and sites with <10 reads and estimates a strand bias, as it handles forward and reverse reads separately. Heteroplasmic variants are called on sites with alternate allele frequencies greater than the default 1%. A maximum-likelihood method adopted from Ye et al. (2014), which factors in sequencing errors, returns a log-likelihood ratio for each heteroplasmic variant to indicate how confident the call is (Ye et al., 2014). It uses 1000 Genomes Phase 3 data as a prior and calculates the posterior probability for each genotype, with the most likely genotype called. The sensitivity in heteroplasmy detection can be altered. Mutserve version 1.1.17 was used for this analysis. A latest version has been released since then (v.2.0.0-rs12).

##### 2.1.3.2 MitoSeek

MitoSeek was released in 2013 and has been used in various disease studies, from melanoma to hepatocellular carcinoma (Guo et al., 2013a; Araujo et al., 2018; Li et al., 2017). Quality control is performed internally to create a report containing statistics like average depth, base quality distribution, and mapping quality distribution. It then filters BAM reads based on these statistics, such as using mapping quality scores ≥20 and base quality scores ≥20. Heteroplasmy detection by MitoSeek works by evaluating the number of raw read counts or the read percentage for an alternative allele. A one-tailed Fisher’s exact test is then used to determine if the rate of heteroplasmic at each site is greater than the default 5% threshold. The sensitivity in heteroplasmy detection can be altered. MitoSeek does not detect homoplasmic variants. MitoSeek version 1.3 was used for this analysis.

##### 2.1.3.3 mitoCaller

mitoCaller is a mitochondrial variant-calling module from the mitoAnalyzer package released in 2015, which has been used in studies on ageing (Ding et al., 2015; Ferrucci et al., 2020). mitoCaller differs from the other tools in its alignment of mtDNA reads. With the mitochondrial genome being circular, mitoCaller proposes a “double alignment” strategy using a conventional rCRS reference and a shifted rCRS reference. A breakpoint is created in the middle of the circular sequence to form a reference that starts at position 8000 and ends at position 7999. The mitoCaller method requires the user to align the sample’s FASTQ reads first against the conventional rCRS reference, and then the FASTQ file is aligned again against the shifted rCRS reference. This creates two BAM files—a conventional mitochondrial BAM and a shifted mitochondrial BAM—which might contain reads that span the traditional start and end base positions. mtDNA variants are called using both BAMs. mitoCaller utilizes a likelihood-based model to predict the genotype at each mtDNA position. Estimation of all possible genotypes is made from the sequence reads; thus, it can call both heteroplasmic and homoplasmic variants. Quality control filters, such as the average sequence depth of the overall mtDNA (>100), base quality scores (≥20), and sequence depth at the calling base position (raw reads ≥40, after base quality score filtering ≥10), are applied to account for the possibility of sequencing errors at each mtDNA position (Ding et al., 2015). The algorithm is based on the ones used in conventional autosomal DNA variant callers but modified to allow for low-heteroplasmic-level allele fractions. MitoCaller version 1.0 was used for this analysis.

##### 2.1.3.4 MToolBox

MToolBox is a complete workflow for mtDNA variant calling (Calabrese et al., 2014). It can accept as input raw read data (FASTQ files) or pre-aligned reads (BAM files). The reads are re-mapped to a user-defined reference (the Reconstructed Sapiens Reference Sequence or the revised Cambridge Reference Sequence) and to the nuclear genome to discard nuclear mitochondrial sequences (NUMTs) and amplification artifacts. Following optional indels re-alignment, the complete mitochondrial genome is reconstructed using genome assembly, and variants are called. The variants are filtered based on the quality scores and read depth and annotated in a VCF file. The reconstructed contig sequences are provided, and haplogroups are assigned. Finally, the variants can be prioritized by taking into account pathogenicity of each mutated allele, determined with different algorithms, the nucleotide variability of each variant site, and occurrence among 1000 Genomes Project samples. MToolBox does not explicitly call homoplasmies but only distinguishes calls when their variant allele frequency is at 100%. All other variants are detected, and their variant allele frequency or heteroplasmic fraction (HF) is reported and can be up to 100%. Homoplasmic variants are identified if their genotype is 1 in the VCF file, which corresponds to HF = 100%. MToolBox by default calls variants with a threshold of HF >20%, but this threshold can be altered by the user. MToolBox version 1.2 was used for this analysis.

##### 2.1.3.5 GATK With HaplotypeCaller (*via* GenotypeGVCFs)

A GATK pipeline with HaplotypeCaller (*via* GenotypeGVCFs) is a commonly used pipeline to identify variants from the nuclear genome. We are evaluating its performance in identifying mtDNA variants, since it is often the standard pipeline applied to all WGS data. Since GATK Haplotypecaller is not a mtDNA caller, a custom script was used to calculate the heteroplasmic level based on the allelic fractions from alternate allele read numbers. Decomposition of multi-allelic variants and normalization were performed with VT v0.57721 (Tan et al., 2015). GATK v3.7 and v4.2 Haplotypecaller were used for this analysis. Recently, the GATK team has introduced guidelines for the identification of mitochondrial short variant discovery that involves the use of Mutect2, a GATK algorithm for somatic variant detection that can be used on mitochondrial mode (Benjamin et al., 2019).

The commands used for alignment and variant calling of the synthetic dataset using all methods are provided in the Supplementary Methods.

#### 2.1.4 Variant Calling Accuracy

To assess variant calling accuracy, a comparison of the results with the variants defining the gold standard was performed (provided in Supplementary Table S7 of Fazzini et al., 2021). Sensitivity, specificity, precision, and F1 scores were calculated for all callers.

### 2.2 Congenital Heart Disease Trio Dataset

The dataset originally consisted of 329 samples from a Congenital Heart Disease (CHD) cohort compiled at the Victor Chang Cardiac Research Institute and was previously used in a study aiming to identify clinically actionable CHD variants (Alankarage et al., 2018). For the purposes of mtDNA variant calling, we identified all samples with in-sample contamination using Haplocheck (Weissensteiner et al., 2021). We removed all trios containing at least one contaminated sample. Our final data consisted of 300 samples forming 126 trios (including trios of siblings with the same parents) mostly of European descent as shown in Alankarage et al. (2018).

#### 2.2.1 Sample Collection and Sequencing

Genomic DNA was extracted as described previously (Szot et al., 2018). DNA sample libraries were prepared using the Illumina TruSeq Nano DNA HT Library Prep Kit and sequenced on the Illumina HiSeq X Ten at Genome.One, Garvan Institute of Medical Research, Sydney, Australia.

#### 2.2.2 Alignment to a Reference Genome

The genomic data were aligned to the 1000 Genomes Reference Genome Sequence (hs37d5) using the Burrows–Wheeler Aligner (BWA) 0.7.17 (Li and Durbin 2009) to create whole-genome BAM files. This reference genome is composed of the Genome Reference Consortium Human Reference 37 (GRCh37) assembly, the revised Cambridge Reference Sequence (rCRS) mitochondrial sequence NC_012920.1, the human herpesvirus 4 type 1, and decoy sequences. We marked duplicates using Picard Tools 2.1.1 (http://broadinstitute.github.io/picard/) and default parameters and then performed local realignment and local recalibration using the Genome Analysis Toolkit (GATK) 3.7 following their “best practices” pipeline (DePristo et al., 2011; Poplin et al., 2017). Mitochondrial-specific BAM files were created using samtools 1.9 software to extract mitochondrial aligned reads (Li et al., 2009; Zhang et al., 2016).

#### 2.2.3 Mitochondrial Variant Calling

The same mtDNA-specific variant callers (Mutserve (Weissensteiner et al., 2016), mitoCaller (Ding et al., 2015), MitoSeek (Guo et al., 2013b), and MToolBox (Calabrese et al., 2014)) were used for our evaluation, and the gold-standard GATK Haplotypecaller “best practices” pipeline (DePristo et al., 2011) (Table 1). Variant calling was performed using the default settings for each caller and standardized heteroplasmy detection evaluations at 1% and 5% threshold. These thresholds were chosen based on the methods’ ideal sensitivity (1% for Mutserve and Mitoseek; >4% for MitoCaller). Again, for mitoCaller and GATK Haplotypecaller, custom scripts were used to filter-returned heteroplasmic variants that met the experimental thresholds (1% and 5%).

#### 2.2.4 Variant Calling Accuracy

A true mitochondrial variant dataset consisting of confirmed variants for the CHD trio dataset was not available to allow us to evaluate the various callers. To determine the accuracy of the callers, an alternative method using the matrilineal inheritance nature of the mitochondria was adopted. As our dataset contained family trios, we compared homoplasmic mtDNA variants identified in the child against homoplasmic mtDNA variants identified in the mother. The resultant shared percentage of homoplasmic variants was used as an indicator of caller accuracy. This approach was used because mtDNA has matrilineal inheritance, so we would more likely see a homoplasmic variant as an inherited variant than a *de novo* homoplasmic variant in offspring, even with the high mutation rate of the mitochondrial genome (Amorim et al., 2019). As an additional metric, we compared heteroplasmic mtDNA variants identified in children against heteroplasmic variants identified in mothers and fathers. Although most heteroplasmic variants occur during lifetime, we still expect a child to share more variants with its mother than its father (Ding et al., 2015; Guo et al., 2013a; Stewart and Chinnery 2015). Rare cases of biparental mtDNA transmission have been reported (Luo et al., 2018) but not replicated by other studies that showed that these findings are related to the presence and inheritance of mega-NUMTs (Balciuniene and Balciunas, 2019; Salas et al., 2020; Wei et al., 2020; Lutz-Bonengel et al., 2021).

#### 2.2.5 Population Frequency of Mitochondrial Variants

We utilized the publicly available HelixMTdb, a database containing a list of variants and their allele frequency in 195,983 unrelated individuals (Bolze et al., 2020), to subset our called variants into rare (≤1% in HelixMTdb) and common (>1% in HelixMTdb).

#### 2.2.6 Pathogenicity Annotation of Mitochondrial Variants

mtDNA variants were annotated using three pathogenicity prediction databases: (1) MitImpact v3.0.1 (Castellana, Rónai, and Mazza 2015), which is a collection of functional impact predictions of all possible mtDNA missense variants, including PolyPhen2, CADD, and APOGEE, a machine-learning-based mitochondrial missense mutation predictor (Castellana et al., 2017); (2) MitoTIP (Sonney et al., 2017), which uses a predictive algorithm that combines known variant history at a position, and a conservation score for the position, to identify regions most vulnerable to pathogenic variants; and (3) MITOMAP (Kogelnik et al., 1998), which contains clinical characteristics associated with mutations, so it provides a library of pathogenic and normal phenotypes.

For this analysis, using the three pathogenicity predictors mentioned above, variants were identified as pathogenic when:

1) MitImpact’s APOGEE prediction for the variant was “P” for pathogenic; or
2) MitoTIP’s prediction score for the variant exceeded the recommended pathogenicity threshold of 12.66 (Sonney et al., 2017); or
3) The variant was marked as “confirmed pathogenic” in MITOMAP.

## 3 Results

### 3.1 Variant Calling Accuracy

**FIGURE 1:**
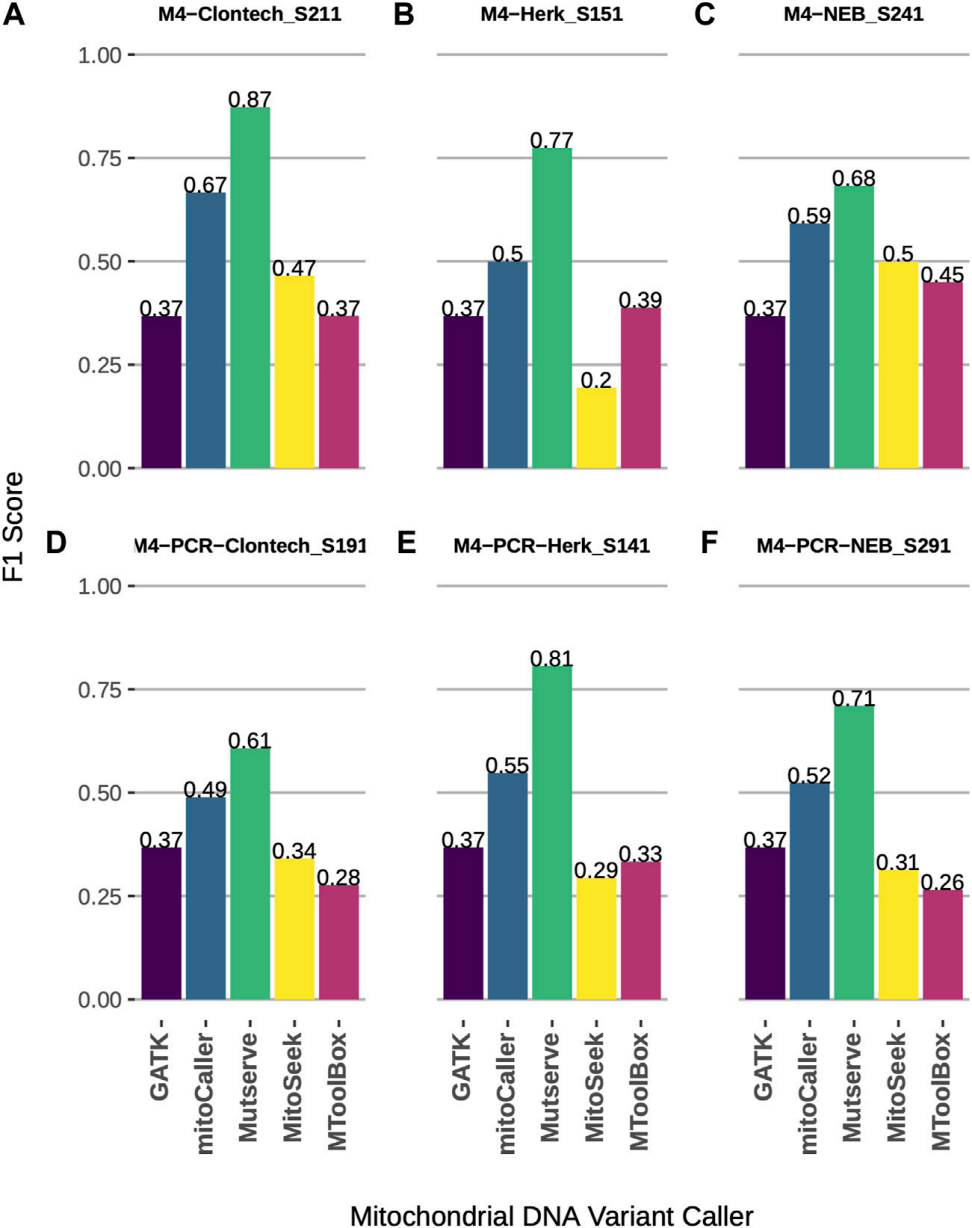
Accuracy comparison of mtDNA variant callers at heteroplasmy threshold of 1%, across different Taq polymerases and DNA extraction protocols using a synthetic benchmark dataset. **(A)** F1 scores of four variant callers using Clontech Taq polymerase and total DNA extraction. **(B)** F1 scores of four variant callers using Herk Taq polymerase and total DNA extraction. **(C)** F1 scores of four variant callers using NEB Taq polymerase and total DNA extraction. **(D)** F1 scores of four variant callers using Clontech Taq polymerase and PCR products. **(E)** F1 scores of four variant callers using Herk Taq polymerase and PCR products. **(F)** F1 scores of four variant callers using NEB Taq polymerase and PCR products.

Using a synthetic gold-standard dataset constructed by the mixture of two mtDNA genomes (Fazzini et al., 2021), we assessed the accuracy of five mtDNA variant callers (Table 1). The level of 1% heteroplasmy and homoplasmy was assessed using all callers. The callers showed comparable performance across three different Taq polymerases and two DNA extraction protocols (Figure 1 and Supplementary Table S1). Mutserve achieved the highest accuracy as measured *via* F1 score (Figure 1, average F1 = 0.74), followed by mitoCaller (average F1 = 0.55), while GATK Haplotypecaller, MitoSeek, and MToolBox had comparable performance with GATK Haplotypecaller performing marginally better when a DNA extraction protocol starting from PCR products was used (Figures 1D–F). Since GATK Haplotypecaller is not a specialized mtDNA variant caller, the number of calls was very low, detecting mostly the homoplasmic variants (nine true-positive and three false-positive calls for all types of Taq polymerases and DNA extraction protocols). This is expected since the level of heteroplasmy assessed was very low (1%) to be detected by a germline variant caller (Supplementary Table S1). The highest call rate was achieved by Mutserve, which also returned the highest number of true positives (Supplementary Table S1). MitoSeek is unable to detect homoplasmic variants, which affects both its call rate and accuracy.

We further explored the methods’ accuracy, using a CHD cohort of families that underwent whole-genome sequencing forming 126 parents–child trios (Alankarage et al., 2018). The average sequencing depth of the mtDNA genome across all samples was 3,887.

To determine the accuracy of each caller, we investigated the percentage of matrilineal inheritance of homoplasmic variants across the study dataset. First, we used the default parameters for each caller (see Table 1 for default heteroplasmy detection thresholds). The total numbers of homoplasmic variants called in the probands of the 126 trios were as follows: 2,630 with mitoCaller, 3,112 with Mutserve, 457 with MToolBox, and 3,357 with GATK Haplotypecaller. The matrilineal inheritance percentages were 87.34%, 95.37%, 61.27%, and 99.46%, respectively (Table 2). Since the default heteroplasmy detection threshold varied so greatly between callers (from 1% to 20%), we applied uniform heteroplasmy detection thresholds of 1% and 5% (Table 3, Supplementary Table S2) to obtain a less biased evaluation. The total number of homoplasmic variants called using a 5% heteroplasmy detection threshold in the probands were 3,147 with mitoCaller, 3,246 with Mutserve, 449 with MToolBox, and 3,357 with GATK Haplotypecaller. The matrilineal inheritance percentages were 99.33%, 98.43%, 60.58%, and 99.46%, respectively (Table 3).

**TABLE 2:** mtDNA variants called in CHD trio dataset by variant callers using their default parameters (1% heteroplasmy threshold for Mutserve, 5% for Mitoseek, 0 for mitoCaller, and 20% for MToolBox).

Caller		Comparing child with parents (126 trios)			Comparing mother with father		
		# Variants in child	# Shared with mother	# Shared with father	# Variants in mother		# Shared with father
GATK Haplotypecaller	Homoplasmy	3,357	3,339 (99.46%)	1,320 (39.32%)	3,348		1,320 (39.43%)
	Heteroplasmy	62	36 (58.06%)	6 (9.68%)	62		6 (9.68%)
mitoCaller	Homoplasmy	2,630	2,297 (87.34%)	980 (37.26%)	2,518		966 (38.36%)
	Heteroplasmy	5,284	2,315 (43.81%)	1,905 (36.05%)	5,551		1,839 (33.13%)
Mutserve	Homoplasmy	3,112	2,968 (95.37%)	1,230 (39.52%)	3,043		1,227 (40.32%)
	Heteroplasmy	1,099	485 (44.13%)	485 (44.13%)	1,441		506 (35.11%)
MitoSeek	Homoplasmy	NA	NA	NA	NA		NA
	Heteroplasmy	160	80 (50.00%)	52 (32.50%)	131		46 (35.11%)
MToolBox	Homoplasmy	457	280 (61.27%)	11 (2.41%)	403		8 (1.99%)
	Heteroplasmy	661	517 (78.21%)	60 (9.08%)	701		64 (9.13%)

**TABLE 3:** mtDNA variants called by variant callers using a 5% heteroplasmy threshold.

Caller		Comparing child with parents (126 trios)			Comparing mother with father	
		#Variants in child	#Shared with mother	#Shared with father	#Variants in mother	#Shared with father
GATK Haplotypecaller	Homoplasmy	3357	3339 (99.46%)	1320 (39.32%)	3348	1,320 (39.43%)
	Heteroplasmy	62	36 (58.06%)	6 (9.68%)	62	6 (9.68%)
mitoCaller	Homoplasmy	3147	3126 (99.33%)	1186 (37.69%)	3139	1,188 (37.85%)
	Heteroplasmy	341	312 (91.50%)	244 (71.55%)	352	248 (70.45%)
Mutserve	Homoplasmy	3246	3195 (98.43%)	1249 (38.48%)	3215	1,249 (38.85%)
	Heteroplasmy	75	29 (38.67%)	0 (0.00%)	90	3 (3.33%)
MitoSeek	Homoplasmy	NA	NA	NA	NA	NA
	Heteroplasmy	160	80 (50.00%)	52 (32.50%)	131	46 (35.11%)
MToolBox	Homoplasmy	449	272 (60.58%)	11 (2.45%)	394	8 (2.03%)
	Heteroplasmy	830	662 (79.76%)	164 (19.76%)	887	164 (18.49%)

In reviewing heteroplasmic variants, we expect to see the offspring inheriting a higher proportion of heteroplasmies from their mothers—although most heteroplasmies arise as *de novo* during lifetime (Ding et al., 2015; Li et al., 2016; Stewart and Chinnery 2015; Guo et al., 2013b). Any sharing of heteroplasmies between fathers and offspring is most likely occurring by chance, as patrilinear inheritance has only been reported once (Luo et al., 2018) but not replicated by other studies (Balciuniene and Balciunas, 2019; Salas et al., 2020; Wei et al., 2020; Lutz-Bonengerl et al., 2021). Hence, we also evaluated the heteroplasmic variants using the matrilineal inheritance comparison. In an ideal scenario, a higher percentage of maternally inherited variants in comparison to variants shared with the father would indicate better accuracy.

The total numbers of heteroplasmic variants called, with each caller’s default parameter, in the probands were 5,284 with mitoCaller, 1,099 with Mutserve, 160 with MitoSeek, 661 with MToolBox, and 62 with GATK Haplotypecaller. The matrilineal inheritance percentages were 43.81%, 44.13%, 50.00%, 78.21%, and 58.06%, respectively (Table 2). At heteroplasmy detection threshold of 5%, the total number of heteroplasmic variants called in the probands were 341 with mitoCaller, 75 with Mutserve, 160 with MitoSeek, and 62 with GATK Haplotypecaller. The matrilineal inheritance percentages were 91.50%, 38.67%, 50.00%, 79.76%, and 58.06.%, respectively (Table 3). The number of heteroplasmic variants called decreased as the heteroplasmy detection threshold increased.

Using only the homoplasmic variant matrilineal inheritance percentage as a metric of accuracy, across all the heteroplasmy detection threshold levels used, the non-mtDNA-specific variant caller GATK Haplotypecaller would be considered the most accurate for homoplasmy calling, followed by mitoCaller and Mutserve. For all callers, the percentage of heteroplasmic variants of the child shared with the mother was always higher than the percentage shared with the father, reflecting our expectation based on matrilineal inheritance. For heteroplasmy threshold of 1%, the percentage of the offspring variants shared with the father was increased for all callers, and the variants shared between the mother and the father. This indicates that at such low heteroplasmy threshold, systematic errors are likely to occur in the same nucleotide positions.

### 3.2 Allelic Distribution of mtDNA Variants

**FIGURE 2:**
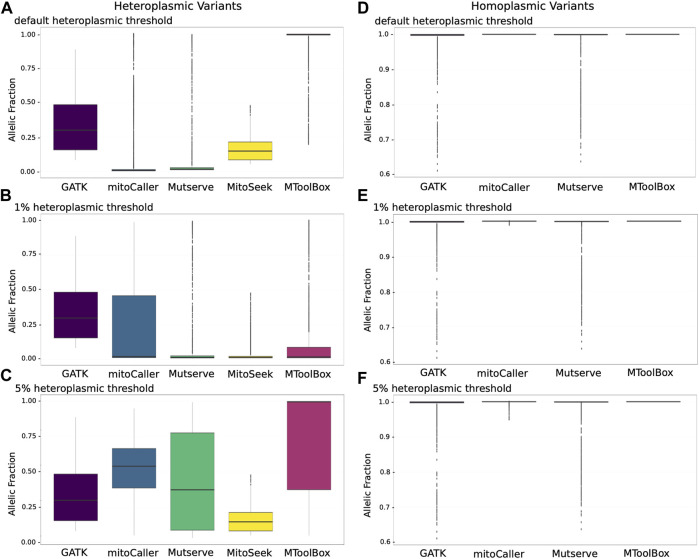
Allelic distributions of mitochondrial variants according to four variant calling methods. **(A)** Allelic fraction distributions of heteroplasmic variants with default detection threshold. The heteroplasmic variant allele fractions for Mutserve, MitoSeek, and MToolBox are at the minimum and equal to their default heteroplasmy thresholds of 1%, 5%, and 20%, respectively. mitoCaller has no threshold, which is reflected in the low VAF values in the plot. With GATK Haplotypecaller, which is not a specific mitochondrial caller, the allelic fraction represents values typical of a heterozygous call from autosomal genomic variant calling (median values: GATK Haplotypecaller—29.9%; mitoCaller—0.5%; Mutserve—1.5%; MitoSeek—4.7%; MToolBox—99.7%). **(B)** Allelic fraction distributions for heteroplasmic variants at a detection threshold of 1% (median values: GATK Haplotypecaller—29.9%; mitoCaller—2.2%; Mutserve—1.5%; MitoSeek—1.5%; MToolBox—1.9%). **(C)** Allelic fraction distributions for heteroplasmic variants at a detection threshold of 5% (median values: GATK Haplotypecaller—9.9%; mitoCaller—54%; Mutserve—37.3%; MitoSeek—14.7%; MToolBox—99.6%). **(D)** Allelic fraction distributions for homoplasmic variants with default detection thresholds and excluding MitoSeek, which does not call homoplasmic variants. As expected for homoplasmic variants, the allelic fractions are almost all at the 100% level. **(E)** Allelic fraction distributions for homoplasmic variants at a detection threshold of 1%. There is no change for GATK Haplotypecaller and Mutserve calls. mitoCaller now includes homoplasmic variants that are in the 99% range (100%—1% heteroplasmy detection threshold). **(F)** Allelic fraction distributions for homoplasmic variants at a heteroplasmy detection threshold of 5%, showing an allele fraction change for mitoCaller to include 95% range.

Due to the variable penetrance of heteroplasmic variants in the mitochondria, we investigated the allelic fraction of all heteroplasmic variants called by the mtDNA variant callers using the CHD trio data analysis (Figure 2). For heteroplasmic variants called using default heteroplasmy detection threshold (Figure 2A), GATK Haplotypecaller variants behaved as expected, with variants restricted to a VAF range of ∼15%–50%, much like a heterozygous VAF for autosomal genomic variants. Mutserve, MitoSeek, and MToolBox heteroplasmic variants had VAFs that started at their default heteroplasmy thresholds of 1%, 5%, and 20%, respectively. mitoCaller had the lowest VAF boundary of close to 0%, which is in line with it having no default heteroplasmy threshold value. MitoSeek seemed to be the only caller that did not call any heteroplasmic variants beyond a 50% VAF. All others called heteroplasmic variants with >75% VAF, with mitoCaller, Mutserve, and MToolBox even calling outlying heteroplasmic variants close to the 100% fraction. The VAF distribution of variants called by MToolBox was skewed towards 100%. This is because MToolBox calls all variants as heteroplasmic and only distinguishing homoplasmic variants when VAF = 100%. As a result, a large number of variants with VAF close to 99% were called as heteroplasmic.

When we set a uniform heteroplasmy detection threshold of 1% (Figure 2B), we saw no change for GATK Haplotypecaller-called variants, as most variants called were well above the 1% detection threshold. For the other callers, the variants’ VAF mostly reflected the 1% threshold level. For mitoCaller, a lot of low-fraction variant calls were removed, while MitoSeek called a higher number of low-fraction variants. MToolBox called a large number of low-level heteroplasmic variants, which altered the VAF distribution towards 1%. At a heteroplasmy detection threshold of 5% (Figure 2C), mitoCaller’s VAF range extended beyond the 50% fraction for heteroplasmic variants, as did that of Mutserve. This was due to the removal of heteroplasmic variants with very low VAFs, which altered the VAF distribution of the remaining heteroplasmic variants called by both callers (Table 3). Interestingly, the VAF distribution of MToolBox variants was not overwhelmed by low-fraction heteroplasmic variants; MToolBox mostly called heteroplasmic variants with very high VAF.

Homoplasmic variants at the default heteroplasmy detection threshold (Figure 2D) were concentrated at the 100% VAF for all callers. GATK Haplotypecaller and Mutserve called homoplasmic variants with VAFs as low as 60%. mitoCaller and MToolBox are both very strict in calling only variants that had a VAF close to 100%, which might explain the high allelic fractions seen in their heteroplasmic variants (Figure 2A). At the 1% heteroplasmy detection threshold (Figure 2E), we saw no distribution change for GATK Haplotypecaller and Mutserve calls compared to the default heteroplasmy detection threshold results. The 1% heteroplasmy detection threshold result for mitoCaller showed a few homoplasmic variants with <100% VAF. At 5% heteroplasmy detection threshold (Figure 2F), there was again no VAF distribution change in the GATK Haplotypecaller and Mutserve calls compared to the 1% threshold results. For mitoCaller, homoplasmic variants were called with VAF as low as 95%. MToolBox still only called homoplasmic variants if VAF = 100%.

### 3.3 Concordance of mtDNA Variants

**FIGURE 3:**
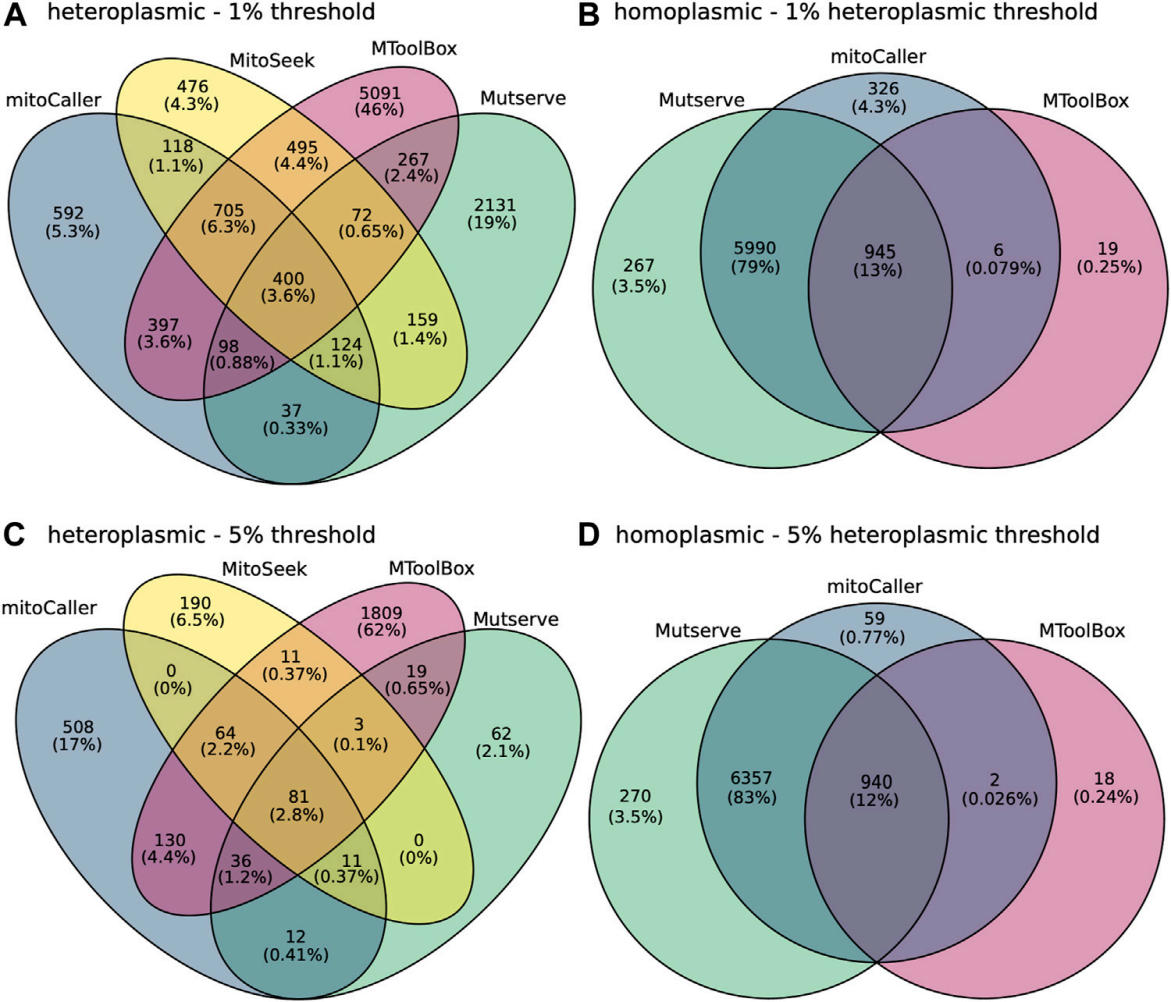
Concordance of mitochondrial variants between the variant callers, Mutserve, MitoSeek, mitoCaller, and GATK Haplotypecaller. **(A)** Concordance of heteroplasmic variants called by the various callers using their default parameters. The first value in the diagram represents the number of mitochondrial variants, and the second value is the percentage of total. **(B)** Concordance of homoplasmic variants called by the various callers using their default parameters, excluding MitoSeek that does not identify homoplasmic variants. **(C)** Concordance of heteroplasmic variants called by the various callers using a heteroplasmic threshold of 5%. **(D)** Concordance of homoplasmic variants called by the various callers using a heteroplasmic threshold of 5%.

Concordance percentages between all four mtDNA variant callers across the 300 samples in our study were calculated for both homoplasmic and heteroplasmic variants using again the CHD trio data analysis (Figure 3 and Supplementary Figure S1). Under default settings, only 45 heteroplasmic variants (representing 0.29% of the total number of variants) were concordant (Supplementary Figure S1A). This low percentage can be attributed to the difference in heteroplasmy thresholds used by the different callers. There is a disproportionately large number of heteroplasmic variants called by mitoCaller that does not have a default heteroplasmy threshold with 68% (10,581) of the variants being unique to mitoCaller, while the percentages of variants unique to other callers were orders of magnitude less (Table 4, Supplementary Figure S1A). There was 13% concordance (919 variants) of homoplasmic variants between calling methods (Supplementary Figure S1B). This low concordance of homoplasmic variants is driven by the low number of variants identified by MToolBox, which distinguishes such calls only when VAF = 100%. At 1% heteroplasmy detection threshold the concordance percentage was 3.6% for heteroplasmic variants (400 variants) and 13% for homoplasmic variants (945 variants) (Figures 3A,B). At the 5% heteroplasmy detection threshold level, we saw 81 concordant heteroplasmic variants representing 2.8% of the total calls (Figure 3C). Mutserve showed a large decrease in the number of heteroplasmic variants calls and MitoSeek. However, the small concordance at this heteroplasmy threshold is again driven by the large number called by both MToolBox and MitoCaller. The concordance of homoplasmic variants decreased to 12% (940 variants) (Figure 3D).

**TABLE 4:** The number of variants called by the mtDNA variant callers for dataset, by variant type, and population frequency.

Heteroplasmy detection threshold	Variant caller	Total variants (300 samples)	Homoplasmic (rare, common)	Heteroplasmic (rare, common)
default	GATK Haplotypecaller	8,019	7,856 (1,456, 6,400)	163 (94, 69)
	mitoCaller	19,387	5,943 (1,029, 4,914)	13,444 (13, 120, 324)
	MitoSeek	360	NA	360 (358, 2)
	Mutserve	10,490	7,202 (1,499, 5,703)	3,288 (3,233, 55)
	MToolBox	2,688	979 (603, 376)	1,709 (1,662, 47)
1%	GATK Haplotypecaller	8,019	7,856 (1,456, 6,400)	163 (94, 69)
	mitoCaller	9,738	7,267 (1,342, 5,925)	2,470 (2,260, 210)
	MitoSeek	2,549	NA	2,549 (2,541, 8)
	Mutserve	10,490	7,202 (1,499, 5,703)	3,288 (3,233, 55)
	MToolBox	8,495	970 (602, 368)	7,525 (7,215, 310)
5%	GATK Haplotypecaller	8,019	7,856 (1,456, 6,400)	163 (94, 69)
	mitoCaller	8,200	6,358 (1,391, 5,967)	842 (796, 46)
	MitoSeek	360	NA	360 (358, 2)
	Mutserve	7,791	7,567 (1,595, 5,972)	224 (204, 20)
	MToolBox	3,113	960 (596, 364)	2,153 (2,083,70)

Note: Population frequency determined using helixMTdb; rare denote variants ≤ population allele frequency of 1%. Common denote variants > population allele frequency of 1%.

Given the strict calling of homoplasmic variants by MToolBox, we investigated the concordance of the rest of callers including GATK HaplotypeCaller in identifying homoplasmic variants and found that to increase to 72%, 85%, and 90% for the default, 1% and 5% heteroplasmy thresholds, respectively. This indicates that the low concordance of these calls is indeed driven by the MToolBox variants.

### 3.4 Classification of mtDNA Variants by Population Frequency and Pathogenicity Prediction

The mtDNA variants called in the CHD trio dataset were categorized using mitochondrial variants population allele frequency dataset HelixMTdb (Bolze et al., 2020) into rare (≤1% frequency in HelixMTdb) and common variants (>1% frequency in HelixMTdb) (Table 4). There were many more common homoplasmic variants than rare variants, as expected. However, a substantial number of variants (>1,000) were in the “rare” category. For heteroplasmic variants, we observed more rare variants than common variants. This may be because there were more *de novo* variants due to the high mutation rate of the mitochondrial genome and accumulation of mutations over a lifetime (Amorim et al., 2019). This was the case regardless of the heteroplasmy detection threshold used. We also saw no large increase in the concordance of variants between callers when we compared the results with only the rare or only the common variants across different heteroplasmy detection thresholds (Supplementary Figures S2–S4).

All variants were annotated with pathogenicity predictions using MitImpact (Castellana, Rónai, and Mazza 2015), MitoTIP (Sonney et al., 2017), and MITOMAP (Kogelnik et al., 1998), based on the criteria described in *Materials and Methods*. For heteroplasmic variants, no common variants were predicted to be pathogenic (Supplementary Table S3). The number of rare variants annotated as pathogenic was the lowest by MITOMAP (1–12 variants), followed by MitoTIP (8–74 variants), and MitImpact (5–815 variants) (Supplementary Table S3). Neither MITOMAP nor MitoTIP predicted any pathogenic variants in the homoplasmic variant set, with only MitImpact predicting pathogenicity for any rare (16–34 variants) or common variants (9–92 variants) (Supplementary Table S4).

## 4 Discussion

When we aim to identify genetic variants from WGS data, be they autosomal or mitochondrial, one of the major considerations in determining which variant caller to use is their accuracy. Here, we provide a systematic evaluation of four mitochondrial variant callers (mitoCaller, MitoSeek, Mutserve, and MToolBox) and a standard germline variant caller included in most current WGS pipelines (GATK Best Practices). We used a publicly available synthetic benchmark mtDNA dataset with known heteroplasmic and homoplasmic variants to determine each caller’s accuracy. We additionally utilized a set of 126 CHD trios and determined each caller’s accuracy based on the mitochondrion’s unique matrilineal inheritance. We also explored the variant properties of each method, using the CHD trio dataset.

Using the synthetic benchmark mtDNA dataset of 1% heteroplasmy mixture and by calculating measures such as F1 score, we found that Mutserve was the most accurate caller for detecting both heteroplasmic and homoplasmic variants. This was the case for all three types of Taq polymerases used in the library preparation process (Clontech, Herk or NEB) and DNA extraction protocol (PCR or PCR-free). This analysis was performed using a uniform 1% heteroplasmy detection threshold for all callers given the expected 1% level of heteroplasmy in the synthetic dataset. Fazzini et al. (2021) that constructed this dataset performed a similar comparison of heteroplasmy detection at different levels by evaluating six variant callers (Mutserve, Vardict, Varscan, Freebayes, GATK, and Lofreq). Apart from Mutserve, the rest of the callers used by Fazzini et al. (2021) were not mtDNA specific but callers that can detect variants with low levels of VAF. Interestingly, when comparing Mutserve’s accuracy for the same data (mixture of 1% heteroplasmy), we found it to be more accurate with average F1 score 0.74. Fazzini et al. (2021) report Mutserve’s accuracy with F1 close to zero. This is the case for all callers they evaluated for this heteroplasmy level. The reason for this discrepancy might be due to the default heteroplasmy detection threshold used by Fazzini et al. (2021), which was 0.4% for all callers. For this threshold, the results are overwhelmed by a very high number of false-positive low-level polymorphic positions, resulting in overall lower accuracy for all callers. This is expected, as most callers were developed to detect higher levels of heteroplasmy. Hence, we believe that an analysis with an expected threshold of 1% provides a more useful evaluation that can inform researchers and clinicians on the accuracy of mtDNA-specific variant callers.

Using the CHD trio dataset and the matrilinear inheritance as a level of accuracy, we found a similarly higher percentage of shared homoplasmic variants (61%–99%) between proband and mother in the called variants for all tools, including GATK Haplotypecaller, which is not a specialized mtDNA caller. This may reflect the more straightforward approach of identifying homoplasmic variants that, in majority, have VAF >60% across all methods. MToolBox exhibited the lowest percentage of matrilinear inherited variants due to the strict calling of homoplasmies only when VAF = 100%. By lowering that threshold to 99%, we found that these percentages increase to 96.59%, 96.22%, and 96.35% for the default, 5%, and 1% heteroplasmy threshold, indicating that potential true-positive calls are missed by such a strict categorization of homoplasmic variants.

We did not expect to see high heteroplasmy matrilineal inheritance rates, since *de novo* variants will occur in both mother and proband as they accumulate during their lifespan. However, since heteroplasmy is reported to be inherited by the mother more often than the father (Stewart and Chinnery, 2015; Guo et al., 2013a), this comparison is still very useful. We found that for higher heteroplasmy thresholds, such as the default thresholds of GATK Haplotypecaller and MToolBox, less heteroplasmic variants were called compared to the other methods, but a higher proportion of these were shared with the mother than the father. This is also in line with our expectation of true-positive calls being shared with the mother.

Using a heteroplasmy threshold of 5%, mitoCaller reported that ∼91.5% of shared heteroplasmic variants were indicative of matrilineal inheritance, a significantly higher percentage than the other callers (Table 3). However, this is most likely due to the higher number of variants mitoCaller calls for this threshold and systematic errors. A higher number of errors returned by MitoCaller is also supported by the fact that the calls made also had a high unexpected patrilinear inheritance (Table 3) and were in their majority unique compared to the other callers (Figure 3).

Using a lower heteroplasmy threshold of 1%, the number of heteroplasmic variants increased threefold for most callers, apart from GATK Haplotypecaller, which is designed to identify variants of higher VAF. At such a low level of VAF, we expect the number of false-positive variants to increase. MToolBox showed the highest number of unique heteroplasmic variants using the CHD trio dataset, indicating potential false-positive calls (Figures 3A,C).

Our exploratory analysis of VAF distributions highlighted the difficulty in classifying heteroplasmic variants (Figures 2A–C). Unlike with autosomal genotyping, which is what GATK Haplotypecaller is designed to perform, there was a large range of allele fractions observed, with mitoCaller, Mutserve, and MToolBox calling heteroplasmic variants with VAFs of up to 99.99%. For homoplasmy, mitoCaller maintained a homoplasmy VAF in line with the heteroplasmy detection threshold. Mutserve was calling homoplasmic variants down to a VAF value of 70%. However, it also called heteroplasmic variants at this same 70% VAF level. This overlap was not clarified when we used different heteroplasmy detection thresholds (Figure 2). Using the default parameters, the VAF distribution of heteroplasmic variants called by MToolBox was skewed towards 100% (median VAF = 99.7%). This is because of a large number of potentially misclassified homoplasmic variants. For the same parameters, the majority of heteroplasmic variants identified by MitoSeek were of higher VAF (median VAF = 14.7%) than the other two specialized mtDNA callers (mitoCaller Median VAF = 0.47%, Mutserve median VAF = 1.5%) (Figure 2A). By modifying the heteroplasmy thresholds, there was a large shift in the VAF distributions with mitoCaller identifying the variants with highest median VAF (for threshold = 5%, median VAF = 54%) apart from MToolBox, which suffers again from high number of potentially misclassified homoplasmic variants (median VAF = 99.6%) (Figure 2C).

The use of different heteroplasmy detection thresholds, from the default values of each method to uniform heteroplasmy detection thresholds of 1% and 5%, has allowed us to fully investigate the impact of this threshold on the variants returned. These results indicate that a higher heteroplasmy detection threshold, such as 5%, leads to greater accuracy in the calling of not only homoplasmic but also heteroplasmic variants. This idea of utilizing a high heteroplasmy detection threshold has also been encouraged by other studies (Ding et al., 2015; Zhang et al., 2016). However, such an approach would eliminate all heteroplasmy with levels <5%—the expected number of false negatives is difficult to estimate.

When investigating the concordance of multiple methods, an assumption is often made that if a variant is called by multiple callers, then it is more likely to be a true variant. Thus, by investigating the balance between the percentage of concordant variants (true variants) and the discordant, or the caller-unique variants (possible false variants) using the real CHD trio data, one could decide on a recommendation of a specific mtDNA variant caller. For the majority of methods (excluding MToolBox), the concordance for homoplasmic variants was high, from 72% using default parameters to 90% for a heteroplasmy detection threshold of 5%. We should note that often systematic artifacts can lead to variants detected by all callers, for example due the capture of recurring translocations of mitochondrial DNA to the nuclear genome, nuclear mitochondrial sequences (NUMTs) present at higher levels.

We saw a very low level of heteroplasmic variant concordance, from 0.29% using default parameters to 3.6% at 1% heteroplasmy threshold between all mt-DNA variant callers, excluding the germline variant caller GATK Haplotype Caller (Figure 3 and Supplementary Figure S1). The true number of heteroplasmic variants in the CHD trio samples is unknown. If the concordant variants are the only true variants in our samples, then the variant callers identify a very large number of false positives. On the other hand, if the majority of the discordant variants are true variants, then that indicates many non-variants (false-negative variants) are being accepted by the various callers. Both scenarios would have important implications in a WGS bioinformatics pipeline aiming to identify disease-causing mutations in mtDNA.

We extended our study to include currently available mitochondrial resources, which might form part of a mitochondrial variant identification pipeline. The CHD trio variants were broken down into rare (≤1%) and common variants (>1%) based on the mitochondrial variant population frequency database, helixMTdb (Bolze et al., 2020) (Table 4). There was a high number of rare variants identified, especially for heteroplasmic variants, for all mtDNA callers. The high mutational rate of mtDNA could be creating many *de novo* variants that are not in HelixMTdb, thereby causing these variants to be flagged as “rare” (Kogelnik et al., 1998; Just et al., 2015; Stewart and Chinnery 2015). The CHD trio dataset is mostly of European descent; therefore, we did not expect any population-specific differences.

A variant identification pipeline could include resources to determine if a variant is pathogenic or not. We annotated both the HelixMTdb rare and common variants using the pathogenicity annotation datasets MitImpact (Castellana et al., 2015), MitoTIP (Sonney et al., 2017), and MITOMAP (Kogelnik et al., 1998). Of the three, MitImpact provided the most pathogenic annotations to variants, since it is a database that contains a prediction for every possible nucleotide change within the 13 gene-coding regions of mtDNA. Both MitoTIP and MITOMAP use known clinical history of existing variants, so this may account for the fewer predictions made by them, compared to MitImpact. A pathogenic annotation from MitoTIP and MITOMAP should be considered more impactful than from MitImpact, as they are supported by functional and clinical evidence.

Many methods have been used to detect heteroplasmy in mtDNA that aim to identify variants with low levels of VAF such as LoFreq (Wilm et al., 2012), Mutect2 (Benjamin et al., 2019), and Varscan2 (Koboldt et al., 2012). However, our analysis has focused on callers that aim to be mtDNA specific, especially since such methods could call both homoplasmic and heteroplasmic variants. Other methods were considered that are mtDNA specific such as Mit-o-matic (Vellarikkal et al., 2015), MitoRS (Marquis et al., 2017), and mitoSuite (Ishiya and Ueda, 2017). We selected methods for evaluation that were publicly available, with a command-line interface and that generate output easily accessible by an NGS pipeline. Finally, we did not include in our comparisons NOVOPlasty (Dierckxsens et al., 2020), which is a *de novo* assembly algorithm and heteroplasmy variant caller that has recently been used more widely.

Our study is one of the first to systematically evaluate mtDNA variant calling algorithms for both heteroplasmy and homoplasmy detection on WGS data. Previous work by Fazzini et al. (2021) has evaluated variant callers that are not mtDNA specific, for heteroplasmy detection only, using only a synthetic dataset and using only a very low heteroplasmy threshold. Other previous work has evaluated ways to analyze the mitochondrial genome using PCR-based enrichment approaches coupled with massively parallel sequencing showing that heteroplasmy detection lower than 15% was possible (Cui et al., 2013) and that high diagnostic sensitivity of mitochondrial diseases can be achieved (Zhang et al., 2012). Although prior to sequencing, other methods existed to test mtDNA (Venegas and Halberg 2012), the consensus recommendation provided by Mitochondrial Medicine Society is to perform massively parallel sequencing or next generation sequencing of the mtDNA genome and should be performed in cases of suspected mitochondrial disease instead of testing for a limited number of pathogenic point mutations (Parikh et al., 2015). Studies have also evaluated the effect of the capture of recurring translocations of mitochondrial DNA to the nuclear genome, known as nuclear mitochondrial sequences (NUMTs) in the analysis of sequencing mtDNA data (Maude et al., 2019; Santibanez-Koref et al., 2019). More recently, studies have provided recommendation in the interpretation (Wong et al., 2020) and prioritization (Bris et al., 2018) of variants detected from NGS. Taken together, our work can provide useful recommendations for mtDNA variant calling from WGS data, a necessary step prior to prioritization and interpretation of variants.

## 5 Limitations of Our Study

A limitation of this study is that the accuracy of each mitochondrial variant caller was to be determined by a small synthetic benchmark mtDNA dataset and using the maternal percentage of homoplasmic variant calls in WGS trios. This measure was used since the true mtDNA variation of the CHD trio data set is not known. Since heteroplasmic variants can occur during lifetime, this measure of matrilineal inheritance is not ideal. It is important for mtDNA variant callers to be accurate not only with homoplasmic calls but also with heteroplasmic ones. With mitochondrial heteroplasmic variants having great variability in VAF levels, which can be as low as 1% or lower in an individual, the calling of heteroplasmic variants is very difficult. Thus, the accuracy of a mitochondrial variant caller in determining heteroplasmic variants is of vital importance. Truly determining heteroplasmic calling accuracy would require greater refinement in heteroplasmy genotyping, especially at low VAFs.

To improve the overall ability to assess mitochondrial variant callers, a large gold-standard mtDNA variant dataset of real human WGS data is still needed that would incorporate both homoplasmic and heteroplasmic variants, at various levels of VAF.

## 6 Conclusion

We have shown that while homoplasmic variant calling from WGS data seems to be consistent between the majority of callers, there remains a significant discrepancy in the calling of heteroplasmic variants. Mutserve showed the best accuracy using a publicly available synthetic benchmark dataset. While GATK Haplotypecaller performed well as a stand-in, it does not have the sensitivity to call the low-level heteroplasmic variants, which the specialized mtDNA variant callers are built for. Since GATK Haplotypecaller is commonly used for WGS analysis, it can be used to detect homoplastic variants without the need for a specialized mtDNA variant caller. However, if heteroplasmy detection is needed, Mutserve showed that it can detect both types of variation with highest accuracy at low levels of heteroplasmy (1%) while maintaining expected levels of matrilinear inheritance. For heteroplasmic variants, we believe that any caller’s result should be treated with scrutiny. To address mtDNA variant annotation, population frequency databases and pathogenicity prediction resources are available now but still need greater development, such as with greater sample numbers, or annotation breakdown by heteroplasmic level, and clinical proof of pathogenicity. We see the study of the mitochondrial genome from WGS data as a developing area of research, with its own peculiarities that do not fit into the same variant discovery pipeline as autosomal variants. The present study provides useful information for building a mtDNA variant pipeline by providing selection metrics for mtDNA variant callers and criteria for the selection of population frequency and pathogenicity annotation datasets. We advocate that caution should be taken when analyzing mitochondrial DNA from WGS data especially when interrogating heteroplasmy at low levels.

## Data Availability

The data analyzed in this study is subject to the following licenses/restrictions: The mitochondrial datasets generated and analyzed during the current study are not publicly available, as they are patient samples and sharing them could compromise research participant privacy. The data may be made available upon reasonable request. Requests to access these datasets should be directed to Sally L Dunwoodie, s.dunwoodie@victorchang.edu.au, and Eleni Giannoulatou, e.giannoulatou@victorchang.edu.au
