# Supplementary material for: Benchmarking the Effectiveness and Accuracy of Multiple Mitochondrial DNA Variant Callers: Practical Implications for Clinical Application
Source: Front Genet. 2022 Mar 8;13:692257. doi: 10.3389/fgene.2022.692257 (PMC8957813; doi:10.3389/fgene.2022.692257)
Supplement: Supplementary file 1 [file DataSheet1.PDF]

Benchmarking the effectiveness and accuracy of multiple Mitochondrial DNA variant callers: practical implications for clinical application

Eddie Ip[1,2], Michael Troup[1], Colin Xu[3], David S Winlaw[4], Sally L Dunwoodie[1,2], Eleni Giannoulatou[1,2,*]

[1]Victor Chang Cardiac Research Institute, Sydney, NSW 2010, Australia

[2]St.Vincent's Clinical School, UNSW Sydney, Australia

[3]School of Computer Science and Engineering, UNSW Sydney, Australia

[4]Heart Institute, Cardiothoracic Surgery, Cincinnati Children's Hospital Medical Centre, Cincinnati, OH, USA

*** Correspondence:**
Eleni Giannoulatou
e.giannoulatou@victorchang.edu.au

Supplementary Material

Supplementary Table S1

Supplementary Table S2

Supplementary Table S3

Supplementary Table S4

Supplementary Figure S1

Supplementary Figure S2

Supplementary Figure S3

Supplementary Figure S4

Supplementary Methods

Supplementary Table 1. Evaluation of all mtDNA variant callers across different Taq polymerases (Herk, Clontech and NEB) and DNA extraction protocols (PCR and PCR-free) using a synthetic gold-standard dataset.

	Caller Statistics				
	TP[1]	FP[2]	FN[3]	Ig[4]	F1[5]
**GATK**					
M4-Clontech_S211	9	3	28	0	0.37
M4-Herk_S151	9	3	28	0	0.37
M4-NEB_S241	9	3	28	0	0.37
M4-PCR-Clontech_S191	9	3	28	0	0.37
M4-PCR-Herk_S141	9	3	28	0	0.37
M4-PCR-NEB_S291	9	3	28	0	0.37
**mitoCaller**					
M4-Clontech_S211	32	27	5	5	0.67
M4-Herk_S151	20	23	17	2	0.50
M4-NEB_S241	29	32	8	3	0.59
M4-PCR-Clontech_S191	23	34	14	6	0.49
M4-PCR-Herk_S141	20	16	17	3	0.55
M4-PCR-NEB_S291	22	25	15	7	0.52
**MitoSeek**					
M4-Clontech_S211	20	29	17	4	0.47
M4-Herk_S151	8	37	29	2	0.20
M4-NEB_S241	23	32	14	2	0.50
M4-PCR-Clontech_S191	14	31	23	4	0.34
M4-PCR-Herk_S141	10	21	27	1	0.29
M4-PCR-NEB_S291	13	33	24	2	0.31
**Mutserve**					
M4-Clontech_S211	31	3	6	4	0.87
M4-Herk_S151	24	1	13	1	0.77
M4-NEB_S241	29	19	8	3	0.68
M4-PCR-Clontech_S191	24	18	13	3	0.61
M4-PCR-Herk_S141	25	0	12	1	0.81
M4-PCR-NEB_S291	27	12	10	5	0.71
**MToolBox**					
M4-Clontech_S211	14	25	23	4	0.37
M4-Herk_S151	13	17	24	1	0.39
M4-NEB_S241	18	25	19	2	0.45
M4-PCR-Clontech_S191	9	19	28	3	0.28
M4-PCR-Herk_S141	10	13	27	1	0.33
M4-PCR-NEB_S291	9	22	28	2	0.26

[1]True Positive; [2]False Positive; [3]False Negative; [4]Ignore; [5]F1 Score

Supplementary Table 2. mtDNA variants called by variant callers using a 1 % heteroplasmy threshold.

Variant Caller		Comparing child with parents (126 trios)			Comparing mother with father	
		#variants in child	#shared with mother	#shared with father	#variants in mother	#shared with father
GATK	Homoplasmy	3357	3339(99.46%)	1320(39.32%)	3348	1320(39.43%)
	Heteroplasmy	62	36(58.06%)	6(9.68%)	62	6(9.68%)
mitoCaller	Homoplasmy	3120	3076(98.59%)	1183(37.92%)	3097	1181(38.13%)
	Heteroplasmy	1023	624(61.00%)	427(41.74%)	1022	410(40.12%)
MutServe	Homoplasmy	3112	2968(95.37%)	1230(39.52%)	3043	1227(40.32%)
	Heteroplasmy	1099	485(44.13%)	485(44.13%)	1441	506(35.11%)
MitoSeek	Homoplasmy	NA	NA	NA	NA	NA
	Heteroplasmy	1040	441(42.40%)	356(34.23%)	1030	319(30.97%)
MToolBox	Homoplasmy	452	276 (61.06%)	11 (2.43%)	399	8 (2.01%)
	Heteroplasmy	2993	1932 (64.55%)	1327 (44.34%)	3058	1326 (43.36%)

Supplementary Table 3. Pathogenicity predictions in rare and common heteroplasmic mtDNA variants, by variant callers.

Heteroplasmy detection threshold	Variant Caller	Rare			Common		
		MITOMAP	MitoTIP	MitImpact	MITOMAP	MitoTIP	MitImpact
default	GATK	1	0	5	0	0	0
	mitoCaller	12	74	818	0	0	0
	MitoSeek	1	0	8	0	0	0
	Mutserve	3	8	41	0	0	0
	MToolBox	0	0	29	0	0	0
1%	GATK	1	0	5	0	0	0
	mitoCaller	3	9	34	0	0	0
	MitoSeek	3	13	39	0	0	0
	Mutserve	3	8	41	0	0	0
	MToolBox	3	9	169	0	0	0
5%	GATK	1	0	5	0	0	0
	mitoCaller	1	0	8	0	0	0
	MitoSeek	1	0	8	0	0	0
	Mutserve	1	0	8	0	0	0
	MToolBox	1	0	33	0	0	0

Note : rare denote variants ≤ population allele frequency of 1%. Common denote variants > population allele frequency of 1%

Supplementary Table 4. Pathogenicity predictions in rare and common homoplasmic mtDNA variants, by variant callers.

Heteroplasmy detection threshold	Variant Caller	Rare			Common		
		MITOMAP	MitoTIP	MitImpact	MITOMAP	MitoTIP	MitImpact
default	GATK	0	0	34	0	0	92
	mitoCaller	0	0	27	0	0	81
	MitoSeek	NA	NA	NA	NA	NA	NA
	Mutserve	0	0	33	0	0	92
	MToolBox	0	0	16	0	0	10
1%	GATK	0	0	34	0	0	92
	mitoCaller	0	0	34	0	0	92
	MitoSeek	NA	NA	NA	NA	NA	NA
	Mutserve	0	0	33	0	0	92
	MToolBox	0	0	16	0	0	9
5%	GATK	0	0	34	0	0	92
	mitoCaller	0	0	34	0	0	92
	MitoSeek	NA	NA	NA	NA	NA	NA
	Mutserve	0	0	34	0	0	92
	MToolBox	0	0	16	0	0	9

Note : rare denote variants ≤ population allele frequency of 1%. Common denote variants > population allele frequency of 1%

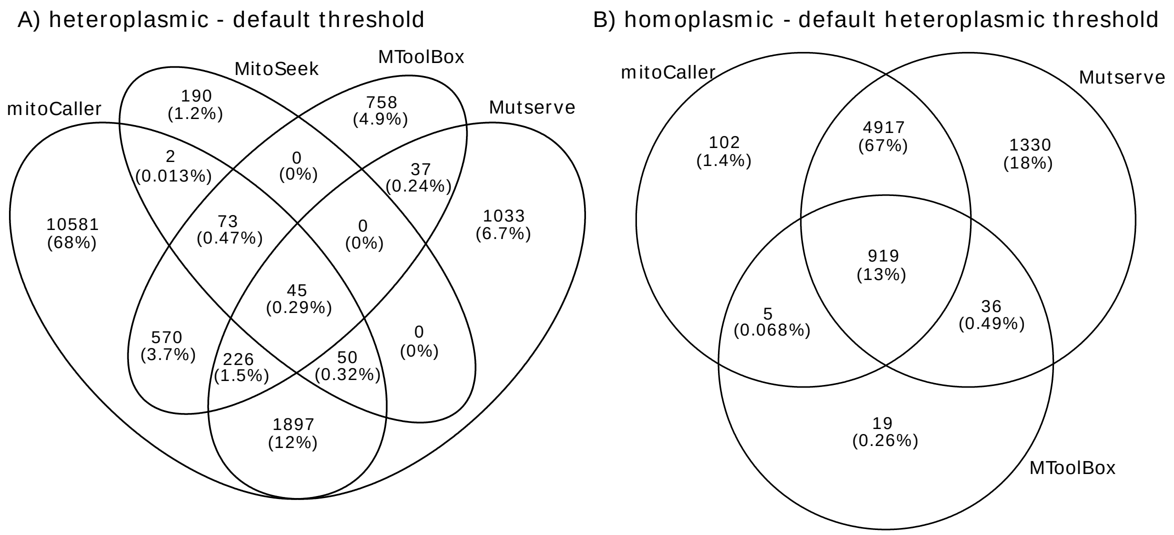

Supplementary Figure 1. Concordance of mitochondrial variants between the variant callers at their default heteroplasmy detection threshold.

(A) Concordance of heteroplasmic variants called by the mtDNA variant callers. **(B)** Concordance of homoplasmic variants called by the mtDNA variant callers.

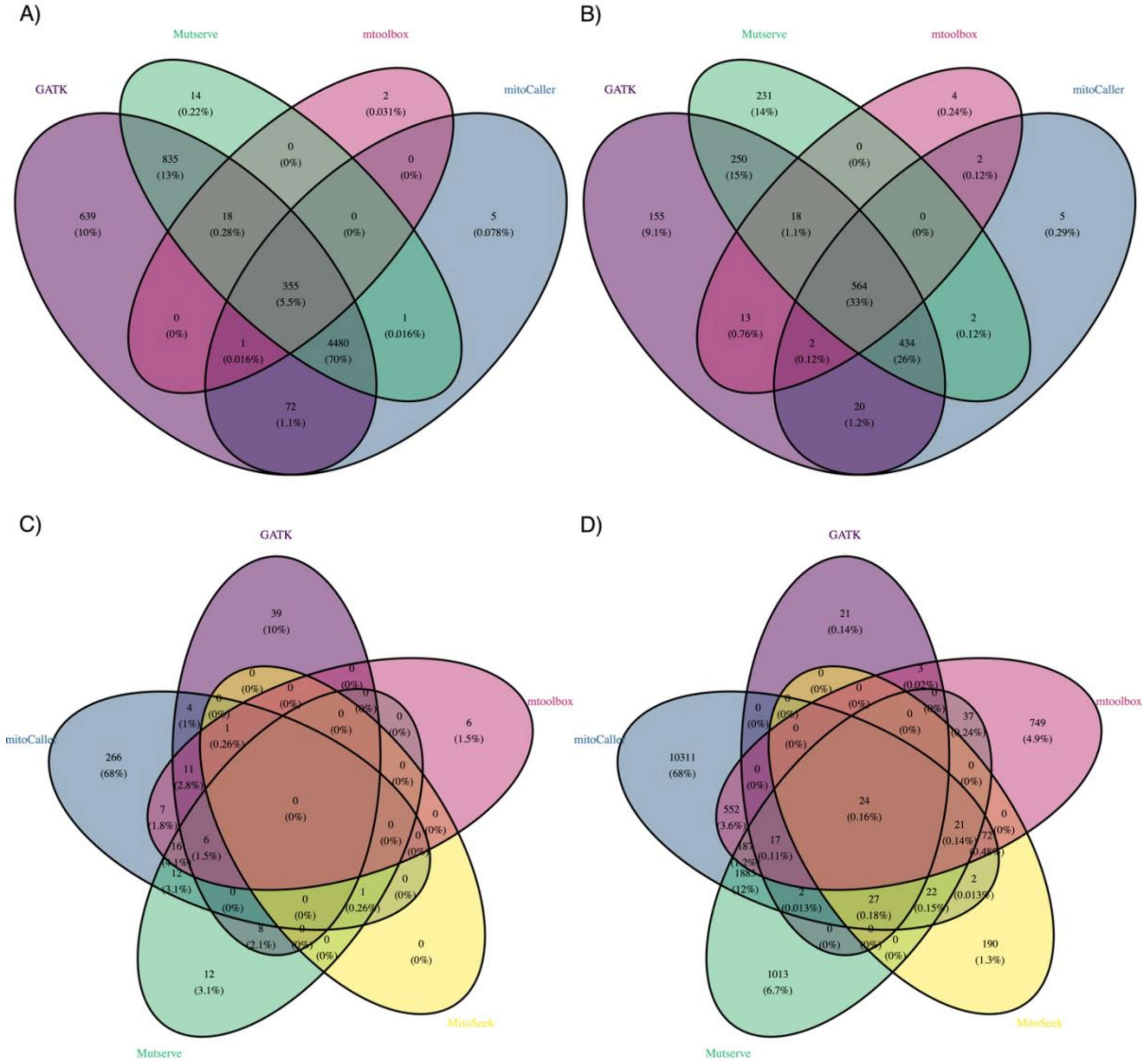

Supplementary Figure 2. Concordance of rare and common mitochondrial variants between the four variant callers using their default heteroplasmy threshold.

Using the utilised the publicly available HelixMTdb, containing allele frequency for 196,554 unrelated individuals, the called mtDNA variants were subset into rare (≤ 1% in HelixMTdb) and common (> 1% in HelixMTdb). **(A)** Concordance of common homoplasmic variants. **(B)** Concordance of rare homoplasmic variants. **(C)** Concordance of common heteroplasmic variants. **(D)** Concordance of rare heteroplasmic variants.

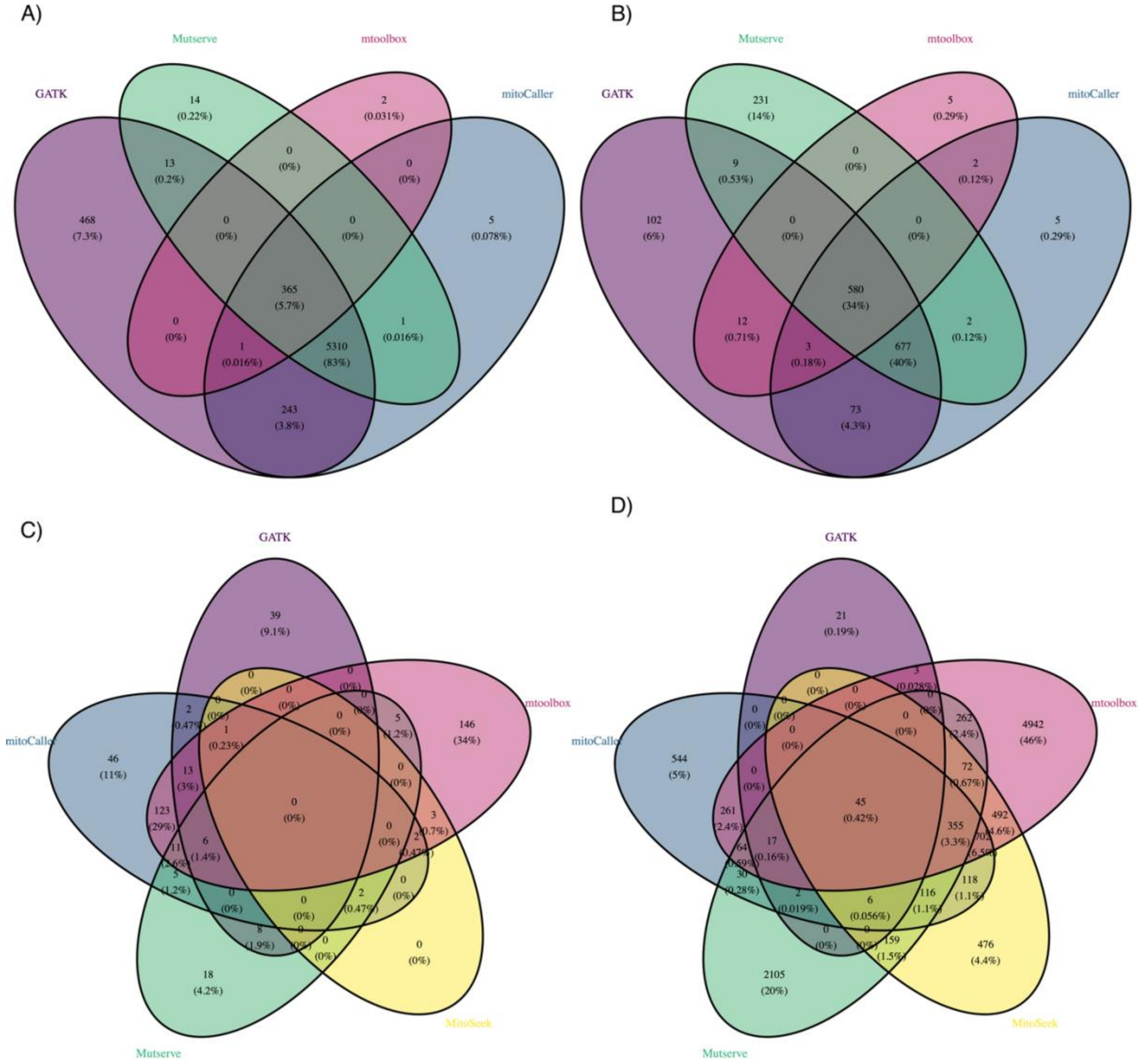

Supplementary Figure 3. Concordance of rare and common mitochondrial variants between the four variant callers using 1% heteroplasmy threshold.

Using the utilised the publicly available HelixMTdb, containing allele frequency for 196,554 unrelated individuals, the called mtDNA variants were subset into rare (≤ 1% in HelixMTdb) and common (> 1% in HelixMTdb). **(A)** Concordance of common homoplasmic variants. **(B)** Concordance of rare homoplasmic variants. **(C)** Concordance of common heteroplasmic variants. **(D)** Concordance of rare heteroplasmic variants.

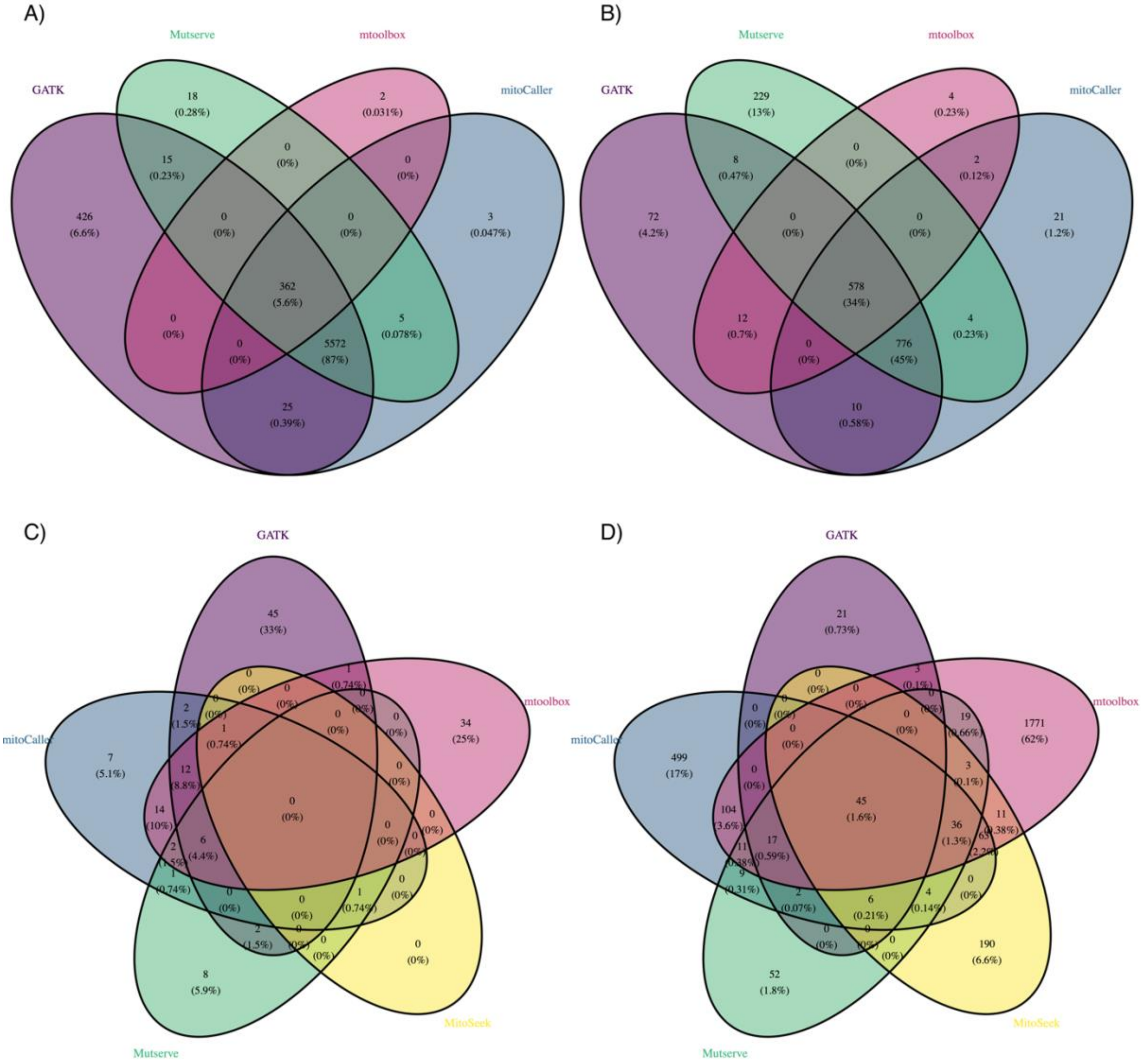

Supplementary Figure 4. Concordance of rare and common mitochondrial variants between the four variant callers using 5% heteroplasmy threshold.

Using the utilised the publicly available HelixMTdb, containing allele frequency for 196,554 unrelated individuals, the called mtDNA variants were subset into rare (≤ 1% in HelixMTdb) and common (> 1% in HelixMTdb). **(A)** Concordance of common homoplasmic variants. **(B)** Concordance of rare homoplasmic variants. **(C)** Concordance of common heteroplasmic variants. **(D)** Concordance of rare heteroplasmic variants.

Supplementary Methods

1. Creation of BAM files from FASTQ files

Alignment

```
$bwa mem -t $NCPUS \
    -R
"@RG\tID:${rg_id}\tLB:${rg_lb}\tPL:ILLUMINA\tSM:${rg_sm}\tPU:${rg_pu
}" \
    "$ref_fasta" $fastq_r1 $fastq_r2 | \
    samtools view -1 - -o "$bam_out"
```

Mark Duplicates

```
${gatk_path} \
    MarkDuplicates \
    INPUT="$in_bam" \
    OUTPUT="$dup_bam" \
    METRICS_FILE="${dup_metrics_filename}" \
    VALIDATION_STRINGENCY=SILENT \
    OPTICAL_DUPLICATE_PIXEL_DISTANCE=2500 \
    ASSUME_SORT_ORDER="queryname" \
    TMP_DIR=$TMPDIR
${gatk_path} --java-options "${java_opt}" \
    SortSam \
    --INPUT "$dup_bam" \
    --OUTPUT /dev/stdout \
    --SORT_ORDER "coordinate" \
    --TMP_DIR $TMPDIR \
    --CREATE_INDEX false \
    --CREATE_MD5_FILE false \
    | \
    ${gatk_path} --java-options "${java_opt}" \
    SetNmMdAndUqTags \
    --INPUT /dev/stdin \
    --OUTPUT "$out_bam" \
    --CREATE_INDEX true \
    --CREATE_MD5_FILE false \
```

```
    --REFERENCE_SEQUENCE ${ref_fasta}
```

2. Commands for all callers

1) GATK

```
# create gvcf
$gatk_path --java-options "$java_opt" \
    HaplotypeCaller \
    -R "$ref_fasta" \
    -I "$in_bam_path" \
    -ERC GVCF \
    -O "$out_gvcf" \
    -L "chrM" \
# "combine" results into database
$gatk_path --java-options "$java_opt" GenomicsDBImport \
    -V "$input_g_vcf" \
    -L "chrM" \
    --genomicsdb-workspace-path "$db_work_path"
# Genotype - final VCF
${gatk_path} --java-options "$java_opt" \
    GenotypeGVCFs \
    -R "${ref_fasta}" \
    -O "${out}" \
    -G StandardAnnotation \
    --only-output-calls-starting-in-intervals \
    --use-new-qual-calculator \
    -V gendb://${db_work_path} \
    -L "chrM"
# decompose and normalise results (e.g. split multi-allelic)
$vt decompose -o "$decomp_vcf" -s "$in_vcf"
tabix -p vcf "$decomp_vcf"
# blocksub
$vt decompose_blocksub -o "$blocksub_vcf" -a "$decomp_vcf"
tabix -p vcf "$blocksub_vcf"
# normalize
$vt normalize -r $ref_fasta -o "$norm_vcf" -n "$blocksub_vcf"
```

```
tabix -p vcf "$norm_vcf"
```

2) mitoCaller

```
#NOTE - requires both the normal reference and a shifted reference - as described in Fazzini

# unshifted output from mitocaller

"$mitocaller" -m -b "$bam_f" -r "$ref_fasta" | grep chrM > "$out_f"

# shifted output from mitocaller - with awk to bring back to equivalent unshifted co-ordinates

"$mitocaller" -m -b "$bam_shifted_f" -r "$ref_fasta_shifted" | grep chrM | \

    awk 'BEGIN{OFS="\t";} {$2 = ($2-16569+7999<=0) ? $2+7999 : $2+7999-16569; print}' | \

    sort -k2 -n > "$shifted_out_f"

# Now replace the first & last 100 lines each of the unshifted output with the adjusted shifted output

head -n 100 "$shifted_out_f" > "$merge_file"

tail -n +101 "$out_f" > "$tmp_f"

head -n -100 $tmp_f >> "$merge_file"

tail -n 100 "$shifted_out_f" >> "$merge_file"

# An awk script was then used to extract all calls that meet the 1% heteroplasmy level.
```

3) Mutserve

```
java "$java_options" -jar "$mutserve_jar" analyse-local \

--input "$in_bam_path" \

--output "$out_f" \

--reference "$ref_fasta" \

--level "$hetMAF"
```

4) MitoSeek

```
ha=0   #heteroplasmy threshold using [int] allele observed, default=0;

hp=1   #heteroplasmy min level (percentage)

# -r --> reference used in BAM files

# -R --> reference used in output files

perl /g/data/a32/Software/MitoSeek/mitoSeek_ed.pl -r rCRS -R rCRS \

    -i $in_path -t 4 -noch -hp $hp -ha $ha -sb 0 -str 4 \

    -samtools "$samtools"
```

5) MToolBox

```
MToolBox.sh -i $config

#Common config file settings:
mtdb_fasta=chrM.fa
hg19_fasta=hg19RCRS.fa
mtdb=chrM
humandb=hg19RCRS
ref=RCRS
MitoExtraction=false
hf_max=0.8
minrd=5

#Settings when hf_min default (0.2):
Hf_min=0.2
UseMarkDuplicates=false
UseIndelRealigner=false

#Settings when hf_min non-default (0.01, 0.05):
hf_min=0.01  #(or hf_min=0.05)
UseMarkDuplicates=false
UseIndelRealigner=false
```
